# Genetic variation and proliferation-promoting virulence function of thymidine kinase from *Paulownia* witches’ broom phytoplasma

**DOI:** 10.1186/s12870-026-09461-2

**Published:** 2026-07-10

**Authors:** Chuansheng Song, Dezhi Kong, Xia Li, Jungang Wang, You Lv, Gaofeng Liu, Lihong Dong, Xiaofei Kang, Xiaofei Li, Keyu Guo, Caili Lin, Cuiping Wan

**Affiliations:** 1 College of Agricultural and Biological Engineering, Heze University, Heze, 274015 China; 2 Key Laboratory of Forest Protection of National Forestry and Grassland Administration, Ecology and Nature Conservation Institute, Chinese Academy of Forestry, Beijing, 100091 China

**Keywords:** *Paulownia* witches’ broom disease (PaWB), Phytoplasmas, Thymidine kinase, Pathogen proliferation, Proliferation-associated pathogenicity factor, Immunoelectron microscopy

## Abstract

**Background:**

*Paulownia* witches’ broom disease (PaWB), caused by phytoplasmas, results in economic losses. Thymidine kinase (TDK) is a key enzyme in the deoxythymidine triphosphate‌ (dTTP) synthesis pathway of phytoplasma, and dTTP serves as an essential precursor for DNA replication during cell proliferation, but its role in regulating phytoplasma proliferation and virulence remains unclear.

**Results:**

To characterize the role of TDK in phytoplasma proliferation and virulence, in this study, two PaWB phytoplasma *tdk* genotypes were identified via sampling in China, and *tdk-1* was the most widely distributed. Phylogenetic analysis revealed that single-copied *tdk* exhibited higher phylogenetic resolution than 16S rRNA for phytoplasma molecular classification. In silico prediction showed that TDK functions as a thymidine kinase in these phytoplasmas. Furthermore, TDK protein expression was detected in PaWB-infected tissues. Immunofluorescence microscopy showed that TDK co-localized with phytoplasmas in sieve tube elements. Immunoelectron microscopy showed that TDK mainly localized in dividing phytoplasma cells. Absolute quantitative analysis revealed that *tdk* mRNA expression was positively associated with phytoplasma proliferation. Overexpressing *tdk* in *Pseudomonas syringae* pv. *tomato* (*Pst*) DC3000 significantly enhanced bacterial growth and increased its virulence in *Arabidopsis*.

**Conclusions:**

Overall, these results identify phytoplasma TDK as a critical proliferation-associated pathogenicity factor that promotes pathogen proliferation, providing a potential molecular target for controlling PaWB.

**Supplementary Information:**

The online version contains supplementary material available at https://doi.org/10.1186/s12870-026-09461-2.

## Introduction

Phytoplasmas (*Mollicutes*) are wall-less bacteria that cause over 1000 plant diseases globally. They are mainly transmitted by insect vectors and colonize phloem sieve elements [1]. *Paulownia* spp., a deciduous tree species belonging to the genus *Paulownia* in the Scrophulariaceae family, is native to China that has been cultivated for over 2,000 years [2]. It is distributed across 25 provinces in China, growing in most areas except for the frigid regions [2]. Moreover, China has more than 1 billion planted paulownia trees, which can provide over 3 million m^3^ of timber annually [3]. Renowned for its fast growth and high-quality lumber, paulownias are used in construction, papermaking, furniture manufacturing, and musical instrument production [2, 4]. *Paulownia* witches’ broom disease (PaWB), caused by phytoplasma, induces witches’ broom, yellowing, stunting, decline, and even death [5]. ‌PaWB‌ is prevalent across all paulownia-growing regions in China, posing a persistent challenge to cultivation [6]. Specifically in Henan Province, where approximately 400 million paulownia trees are planted, over 3 million young trees die from the disease each year, resulting in an annual reduction of about 200,000 m^3^ in the growth volume of diseased trees [7, 8]. These PaWB phytoplasmas have been investigated for more than 50 years [5]. However, the pathogenic mechanisms, particularly the molecular processes underlying disease symptoms, are still not fully understood. Previous studies have mainly focused on phytoplasma effectors, which are secretory virulence factors that hijack host cellular machinery, disrupting development and suppressing immunity [1, 9]. The pathogenicity of non-secreted phytoplasma proteins remains limited, primarily due to the inability to culture phytoplasmas in vitro and the lack of phytoplasma genetic transformation systems.

Symptoms of phytoplasma infection only appear after bacterial titers surpass a critical threshold [10]. Their proliferation-driven pathogenicity relies on efficient DNA replication, supported by the nucleotide salvage pathway, which compensates for the absence of *de novo* synthesis genes [11]. Thymidine kinase (EC 2.7.1.21; TK in eukaryotes and viruses, TDK in prokaryotes) is the rate-limiting enzyme in this process [12]. TDK is a Mg^2+^-dependent enzyme that catalyzes the phosphorylation of 2’-deoxythymidine to deoxythymidine monophosphate, initiating the cascade that produces deoxythymidine triphosphate (dTTP), which is required for DNA replication and repair [13].

There are two evolutionarily conserved isoforms of TK: cytosolic TK1, which is universally present across all life forms, and mitochondrial TK2, which only functions in animals [14]. In human cells, TK1 plays a crucial role in cellular proliferation and the regeneration of thymidine nucleotides within the DNA salvage pathway [13, 15–17]. The CDK4/6–Rb–E2F pathway drives G1 to S phase progression by activating the transcription of S-phase genes, including TK1 [18]. Therefore, the transcript level of *TK1* gene peaks during the G1/S transition and S phase [15]. Previous studies have shown that TK1 promotes tumor progression by mediating cell proliferation in pan-cancer [15]. In tumor cells, mutations or deletions in the genes regulating TK1 lead to its persistent activation, resulting in continuous cell proliferation [18]. This correlation with malignancy makes TK1 a prognostic biomarker and a therapeutic target in cancer [15]. In *Arabidopsis thaliana*, double mutants lacking both *TK1* isoforms (*AtTK1a* and *AtTK1b*) are embryonically lethal, whereas single mutants display normal phenotypes, highlighting the essential role of TK1 in plant development [19]. The replicative importance of thymidine kinase has also been shown in viral pathogens. For instance, α-herpesviruses depend on TK activity for lytic replication and to establish latent infections. Consequently, TK-deficient strains have severely attenuated or no virulence [20]. Although the role of bacterial TDK in virulence remains unknown, mutations disrupting repressors of purine and pyrimidine biosynthesis in *Staphylococcus aureus* enhance its virulence [21, 22], suggesting that increased purine/pyrimidine levels promote bacterial pathogenicity.

Parallels across kingdoms introduce the hypothesis that phytoplasma TDK acts as a master regulator of bacterial proliferation and disease progression through its role in dTTP salvage. To test this hypothesis, we quantified *tdk* mRNA copies per phytoplasma cells and phytoplasma load from PaWB samples of varying severity, clarifying their relationship. Immunoelectron microscopy further established the relation between phytoplasma proliferation and its TDK protein level. Since phytoplasmas are unculturable in vitro and lack a genetic transformation system, we overexpressed TDK in *Pst* DC3000 to confirm its role in pathogen proliferation and virulence. This work will lay a foundation for developing targeted drugs to inhibit phytoplasma reproduction and control the disease.

## Materials and methods

### PaWB-infected and healthy plants

PaWB-infected leaves of paulownias were collected from different regions in China using simple random sampling, air-dried with color-changing silica gel, and stored in glass desiccators at 25 °C, to reveal *tdk* gene polymorphisms in PaWB phytoplasmas (Table S1). A total of 87 Samples were collected using ‌high-reach pruner‌ from 48 districts or counties across 15 provinces or municipalities in China, with 1 to 5 samples gathered per site. The geographic distribution and genotypic composition of PaWB *tdk* samples in China were visualized using ArcGIS 10.8.2. The visualized distribution map was constructed using the standard map from the Standard Map Service website of the Ministry of Natural Resources of the People’s Republic of China, with approval number GS (2020) 4619, and the boundary of the base map has not been altered.

Fresh young stems were collected from three PaWB-infected and three healthy paulownia trees in a field in Heze City, Shandong Province, China, for immunofluorescence microscopy analysis. Each tree contributed one stem sample (*n* = 3 per group). PaWB-infected and healthy tissue-cultured paulownia plantlets were used in vitro to determine the phytoplasma concentration and perform mRNA expression, immunoblotting, immunofluorescence microscopy and immunoelectron microscopy analyses.

### Sequence polymorphism analysis of PaWB *tdk* genes

Total DNA from PaWB-infected and healthy dried leaves of paulownias was purified using a rapid plant genomic DNA extraction kit (DN14; Aidlab, Beijing, China) following the manufacturer’s protocol. A primer pair, tdk-upF/tdk-dnR (Table S2), was designed based on the *tdk* gene sequences of onion yellow phytoplasma (GenBank: AP006628) and aster yellow witches’ broom phytoplasma (GenBank: CP000061) and was used to amplify a fragment by PCR, spanning 82-bp upstream of the initiation codon and 83 bp downstream of the termination codon of the *tdk* gene. PCR amplification was performed in 25 µL volumes containing 0.5 µL (10 µM) of each primer, 12.5 µL of 2 × Taq PCR Mix (Tiangen Biotech, Beijing, China), 10.5 µL of double distilled water, and 1 µL of the undiluted DNA. The amplification protocol was performed as follows: initial denaturation at 94 °C for 4 min; 35 cycles of 94 °C for 30 s, 53 °C for 30 s, and 72 °C for 60 s; final extension at 72 °C for 5 min. The PCR products were sequenced using the above primer pair and assembled using software package DNAMAN 6.0. The experiments were repeated three times. Open reading frame (ORF) sequences of the *tdk* genes from different PaWB phytoplasma strains were aligned using DNAMAN 6.0, revealing *tdk* genotypes and sequence polymorphisms. The *tdk* gene sequences were submitted to GenBank via the NCBI Bankit online tool.

### Bioinformatics analysis

Sequence analysis and alignment were performed using DNAMAN 6.0 software. A phylogenetic tree was constructed using MEGA 7.0 software [23] via the neighbor-joining method with 1000 bootstrap replicates [24, 25] and visualized and refined using the ChiPlot online platform [26]. The molecular weight of the protein was predicted using ProtParam tool of ExPASy 3.0 [27]. Signal peptides were identified using SignalP 5.0 [28], and subcellular localization was determined using Cell-PLoc 2.0 [29]. Functional domains were analyzed using NCBI CD-search tools.

### Protein secretion activity analysis

The *Escherichia coli* alkaline phosphatase (phoA) gene fusion assay was performed as previously described [30]. In our previous study, phoA and signal peptide-deleted phoA (mphoA) were cloned into the pET28a (+) vector, generating pET-phoA and pET-mphoA, respectively [30]. In the present study, the *tdk-1* and *tdk-2* ORFs from PaWB were amplified using primer pairs tdk1F-mphoA/tdkR-mphoA and tdk2F-mphoA/tdkR-mphoA (Table S2) and fused to pET-mphoA, resulting in the pET-*tdk-1*-mphoA and pET-*tdk-2*-mphoA constructs. Three constructs (pET-*tdk-1*-mphoA, pET-*tdk-2*-mphoA, and positive control pET-phoA) were transformed into *E. coli* BL21(DE3) and plated on LB agar containing 90 ng/mL BCIP, 0.1 mM IPTG, 75 mM Na_2_HPO_4_, and 50 µg/mL kanamycin. Blue colonies indicated successful PhoA secretion after incubation at 37 °C overnight, and white colonies indicated an inactive enzyme. The experiments were repeated three times.

### Molecular docking and molecular dynamics simulation

The 3D structure of *tdk* was predicted using AlphaFold v3.0.1, and the molecular energy was minimized with SYBYL-X 2.1.1. Molecular docking studies were conducted using AutoDock Vina v1.2.5 [31], and the ligand complex structure was visualized and generated using Discovery Studio 2019 and PyMOL 3.0. Molecular dynamics simulations and root mean square deviation calculations were performed using the GROMACS 2023.3 software package.

### Expression and purification of PaWB TDK proteins

The ORFs of the *tdk-1* and *tdk-2* genes were amplified from PaWB DNA using primer pairs tdk1F-per28a/tdkR-pet28a and tdk2F-per28a/tdkR-pet28a, respectively (Table S2), and the PCR products were purified. The pET-28a (+) plasmid was linearized via BamHI/XhoI digestion (Takara Bio, Beijing, China) and purified. Fragment–plasmid recombination was performed using an In-Fusion HD Cloning Kit (Takara Bio, Beijing, China), followed by transformation into *E. coli* DH5α (Tiangen, Beijing, China). Positive transformants were selected on Luria Bertani (LB)-kanamycin (50 µg/mL) plates and validated by sequencing. Recombinant plasmids pET28a-*tdk-1* and pET28a-*tdk-2* were transformed into *E. coli* BL21(DE3) (TIANGEN, Beijing, China). Single colonies were grown in LB-kanamycin (50 µg/mL) at 37 °C to an optical density at 600 nm (OD_600_) of 0.4, induced with 0.1 mM isopropyl β-D-1-thiogalactopyranoside (IPTG), and incubated with shaking at 140 rpm for 8 h at 28 °C before harvest. The cell pellet was obtained by centrifugation and stored at -20 °C until analysis.

Protein purification was performed by resuspending a 0.5-g cell pellet in 40 mL lysis buffer (50 mM Tris-HCl, 0.5 M NaCl, 5% glycerol, 10 mM imidazole, 1% Nonidet P-40, 0.25% Tween-20, 1 mg/mL lysozyme, 25 µg/mL RNase A, 10 µg/mL DNase I, 1mM EDTA-free protease inhibitor, pH 8.0). Then, samples were vertexed and incubated on ice for 2 h with vertexing every 30 min. After centrifugation (12,000 × g, 30 min, 4 °C), the supernatant was loaded onto a Ni-IDA 6 FF column (2 mL resin in 15 mL column, Sangon Biotech, Shanghai, China) pre-equilibrated with lysis buffer. The column was washed with 60 mL of lysis buffer and eluted in 4-mL of elution buffer (50 mM Tris-HCl, 0.5 M NaCl, pH 8.0) containing 10, 20, 50, 60, 100, 150, 200, 250 and 400 mM imidazole. Fractions were analyzed using sodium dodecyl sulfate-polyacrylamide gel electrophoresis (SDS-PAGE), as described by Ji et al. [32] and stored at − 80 °C. The anti-TDK polyclonal antibody was generated using purified TDK protein by Zoonbio Biotechnology (Nanjing, China).

### Immunoblotting

Proteins were separated by SDS-PAGE using 5% stacking and 12% resolving gels and transferred to polyvinylidene fluoride membranes (90 min). Membranes were blocked overnight at 4 °C with 5% skim milk in tris buffered saline with tween-20 (TBST) and then washed three times in TBST for 15 min each time. Membranes were incubated with rabbit anti-PaWB TDK polyclonal antibody (1:200 in TBST, 25 °C, 1 h) and washed three times in TBST (15 min/wash). Membranes were incubated with alkaline phosphatase-conjugated mouse anti-rabbit IgG (1:1000 in TBST, 25 °C, 1 h). Following the final washes (3 × 15 min TBST), protein signals were detected using 5-bromo-4-chloro-3-indolyl phosphate / Nitro Blue Tetrazolium substrate. The experiments were repeated three times.

### Immunofluorescence microscopy

Transverse or longitudinal hand sections (~ 100 μm thickness) from fresh stem segments of healthy and diseased tissue-cultured and field-grown paulownias were fixed in 95% ethanol (30 min), washed three times with phosphate-buffered saline (PBS), and incubated with anti-TDK primary antibody at 37 °C for 1 h. After three washes with PBS containing 0.1% Tween-20 (PBST), the segments were incubated with fluorescein isothiocyanate‌ (FITC)-conjugated goat anti-rabbit IgG secondary antibody (37 °C, 1 h) and washed three times with PBST. To discriminate TDK-derived fluorescence from plant autofluorescence, sections were counterstained with 10 mg/mL 4’6-diamidino-2-phenylindole (DAPI) in PBS–glycerol mounting medium, confirming the presence of a phytoplasma in phloem tissues at FITC-positive sites. Sections were analyzed using an Olympus IX73 fluorescence microscope (Tokyo, Japan) with dual-channel excitation (DAPI: 358 nm UV; FITC: 495 nm). Paired images from identical fields were sequentially captured for each fluorophore. Image overlays were generated using ImageJ software 1.8 to verify the colocalization signals. The experiments were repeated three times.

### Immunoelectron microscopy for subcellular localization

Fresh tissue-cultured stem segments (3 × 1 × 1 mm) of paulownia plantlet were fixed with 2% glutaraldehyde/1% osmium tetroxide, dehydrated in an ethanol series (70, 80, 90, 95 and 100%), and embedded in Epon812 resin (Hedebio, Beijing, China). Ultrathin Sect.  (70 nm) collected on Formvar-coated nickel grids (3 mm) were sequentially treated with PBS–glycine, washed with PBGT, incubated with the primary antibody (TDK antibody, 1:800 in PBST, 1 h at 25 °C), rinsed in PBGT, incubated with the colloidal gold-conjugated goat anti-rabbit IgG secondary antibody (in PBST, 1.5 h at 25 °C), successively washed with PBGT, PBGT–glycine, PBS, and distilled water, signal-enhanced (15 min in darkness), stained with uranyl acetate (15 min), and rinsed in distilled water. Samples were examined using a Hitachi H-7500 transmission electron microscope (80 kV, Tokyo, Japan) with digital image acquisition.

### Absolute quantification of phytoplasma load and *tdk* transcript abundance

An absolute quantification method was established to determine the phytoplasma load and *tdk* mRNA transcript abundance while eliminating interference from trace DNA contamination in RNA samples. Root-free paulownia tissue-cultured plantlet were homogenized in liquid nitrogen, and total DNA and RNA were extracted using DN14 and RN53 kits (Aidlab, Beijing, China), respectively. Reverse transcription was performed using TransScript^®^ One-Step gDNA Removal and cDNA Synthesis SuperMix (AT311-02, TransGen Biotech, Beijing, China), with experimental (with enzyme) and control (enzyme replaced by double distilled water) reactions used to account for residual DNA. The *tdk* gene fragment amplified by primers tdk1F-pet28a/tdkR-pet28a (Table S2) was gel-purified as the quantification standard, and the DNA copy number was calculated according to a previously described method [33]. Ten-fold serial dilutions of the standard were used to generate the calibration curve (y = − 3.5457x + 41.492, R^2^ = 0.9965; x: log10 [copy number], y: Ct value). Quantitative PCR was conducted on DNA extracts, cDNA products, and standard dilutions with primers tdk-RT2F/tdk-RT2R (Table S2) to calculate the phytoplasma abundance per gram of tissue and *tdk* mRNA copies per phytoplasma cell. The experiments were repeated three times.

### Bacterial inoculation assay in *Arabidopsis thaliana*

A bacteria inoculation assay was performed as previously described [34]. The PaWB *tdk-1* and *tdk-2* ORFs were amplified using primer pairs tdk1F-pHM1/tdkR-pHM1and tdk2F-pHM1/tdkR-pHM1 (Table S2) and cloned into the pHM1 vector to generate the pHM1-*tdk-1* and pHM1-*tdk-2* plasmids [34]. Rosette leaves from 4- to 5-week-old wild-type *A. thaliana* were infiltrated with *Pst* DC3000 containing pHM1, pHM1-*tdk-1*, or pHM1-*tdk-2* plasmids at an OD_600_ of 0.001–0.002. Representative leaves were sampled and photographed at 3 days post-inoculation (dpi). Leaf discs were collected, homogenized, serially diluted in sterile H_2_O, and plated on Luria-Marine medium with 50 µg/mL rifampicin at 28 °C for 12–18 h. Bacterial colonization was calculated as the number of colony-forming units (CFUs/cm^2^) and represented on a logarithmic scale. The experiment was repeated three times.

### Statistical analysis and visualization

Significant difference and two factor correlation analyses were conducted using SPPS 26.0 software. Statistical analyses between two groups were performed using Student’s t-test. Differences among three or more groups were assessed by one-way ANOVA followed by Tukey’s test. Chart and scatterplots were generated using Microsoft Excel 2024. Before performing one-way ANOVA, data normality was assessed using the Shapiro-Wilk test and homogeneity of variances was verified using Levene’s test. All datasets satisfied these assumptions. For two-group comparisons, Student’s *t*-test was applied after confirming normality and, where appropriate, equality of variances. Data are presented as mean ± standard error from three biological replicates.

## Results

### PaWB *tdk* genotypes exhibit different geographical distribution patterns across China

**Fig. 1:**
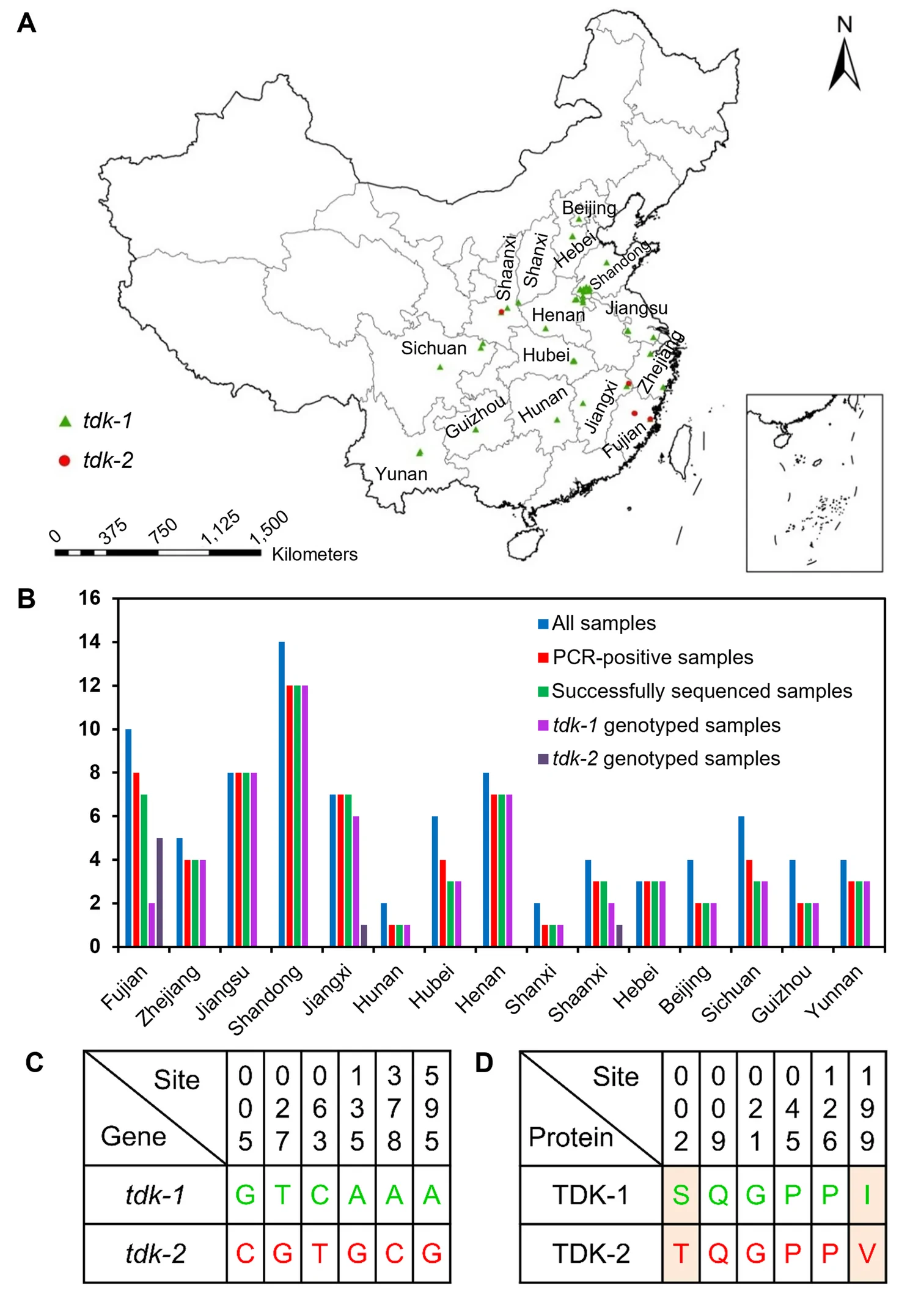
Variations and genotypic geographic distribution of PaWB *tdk* genes. **A** Geographic distribution and genotypic composition of sampled PaWB *tdk* in China. **B** Number of samples across Chinese provinces. **C** Nucleotide variation in the ORFs of *tdk-1* and *tdk-2* genes. Numbers in the ‘Site’ row indicate nucleotide variation sites within the ORFs of *tdk* genes. A, C, G, and T represent adenine, cytosine, guanine, and thymine, respectively. **D** Amino acid variations between TDK-1 and TDK-2 proteins. Numbers in the ‘Site’ row indicate amino acid positions encoded by the six nucleotides in figure (**B**). The G/C mutation at nucleotide position 005 results in the substitution of Ser/Thr at amino acid position 002. The A/G mutation at nucleotide position 595 results in the substitution of Ile/Val at amino acid position 199. G, I, P, Q, S, T, and V represent glycine, isoleucine, proline, glutamine, serine, threonine, and valine, respectively

We conducted nationwide sampling across 48 counties or districts in 15 provinces or municipalities in China to assess the genotypic diversity of PaWB *tdk* (Fig. 1A, B; Table S1). A total of 87 samples were subjected to PCR, of which 66 were successfully sequenced (Fig. 1B, Table S1). Two distinct *tdk* genotypes were identified: *tdk-1* (GenBank: OP913234) and *tdk-2* (GenBank: OP913235). *tdk-1* was identified in 59 samples, accounting for 89.4% of the total. In contrast, *tdk-2* was only detected in Fujian, Jiangxi, and Shaanxi Provinces, with 7 samples, representing 10.6% of the sequenced isolates (Fig. 1A, B; Table S1). Both genotypes contained 630-bp ORFs with 6 nucleotide polymorphisms (Figs. 1C, S1). Protein translation showed that thymidine kinase paralogs differed in two amino acid substitutions (Ser/Thr_2_ and Ile/Val_199_) (Figs. 1D, S1), suggesting functional divergence between genotypes. In summary, two *tdk* genotypes were identified via nationwide sampling, with *tdk-1* being the most widely distributed.

### Single-copy *tdk* exhibits higher phylogenetic resolution than 16S rRNA for phytoplasma molecular classification

**Fig. 2:**
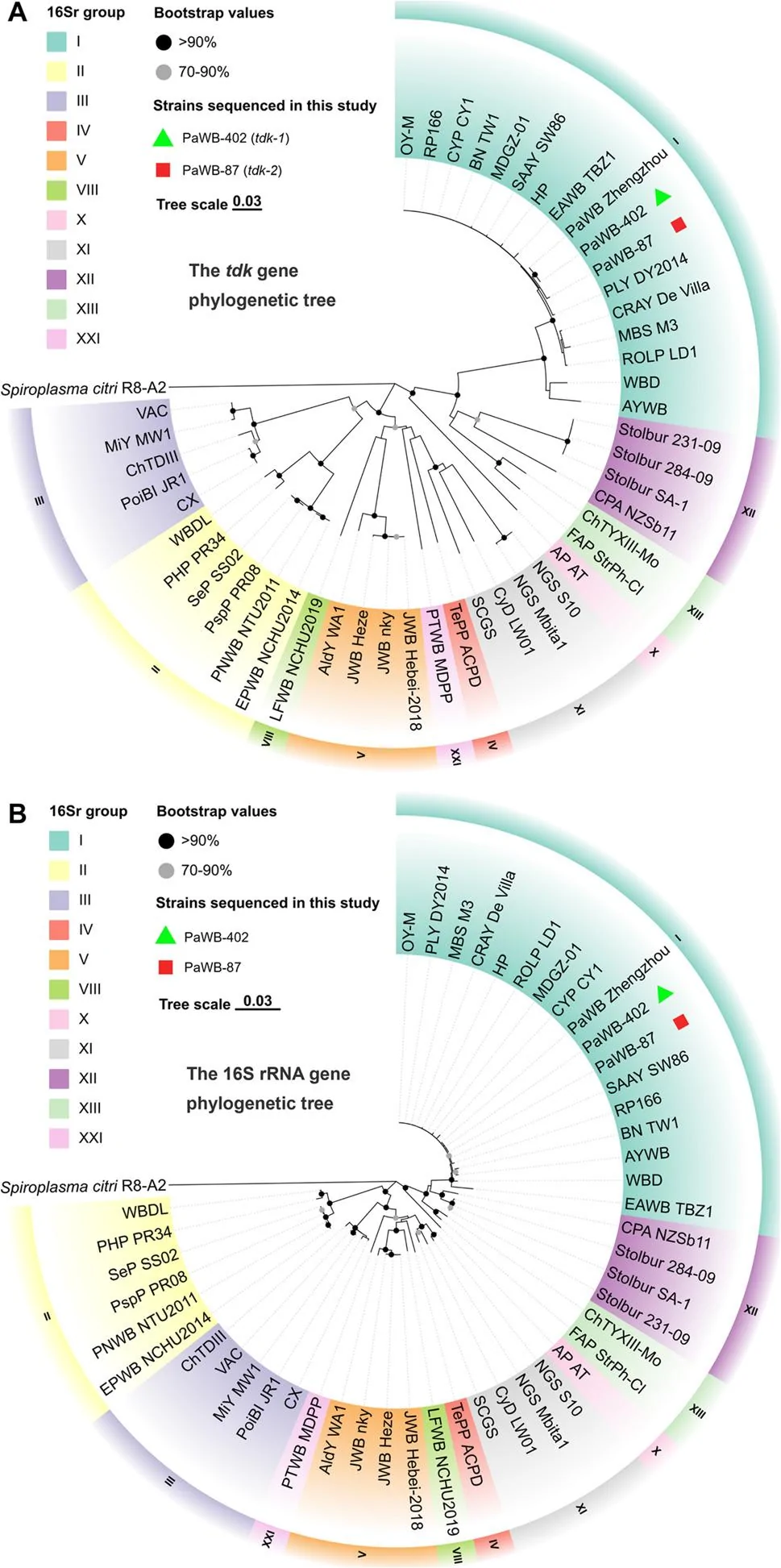
Phylogenetic relationships of phytoplasmas. Phylogenetic trees were constructed using the neighbor-joining method with *tdk* (**A**) and 16S rRNA (**B**) sequences from phytoplasmas and *Spiroplasma citri* (outgroup), respectively. Topological support was assessed with 1000 bootstrap replicates. Locus tag in the genome of *tdk* and 16S rRNA of the reference strains are shown in Table S3. Green triangles and red squares represent the strains sequenced in this study. Strain PaWB-402 carries the *tdk-1* gene, and PaWB-87 carries the *tdk-2* gene

We built and compared phylogenies using *tdk* and 16S rRNA to clarify the evolutionary relationships and taxonomic utility of *tdk* in phytoplasmas. The dataset included 46 published phytoplasma sequences from GenBank with *Spiroplasma citri* as the outgroup (Table S3). The two phylogenetic trees showed a consistent topology (Fig. 2A, B). However, the *tdk*-based tree (Fig. 2A) had longer branches and appeared more dispersed than the 16S rRNA tree (Fig. 2B), indicating a higher genetic variability in *tdk*. *tdk* was a single-copy gene in all examined phytoplasma genomes, but 16S rRNA consistently appeared as dual copies. These results indicate that *tdk* offers superior resolution for phytoplasma classification due to its higher substitution rate and stable copy number compared to 16S rRNA.

### In silico prediction of TDK’s function as a thymidine kinase in phytoplasmas

**Fig. 3:**
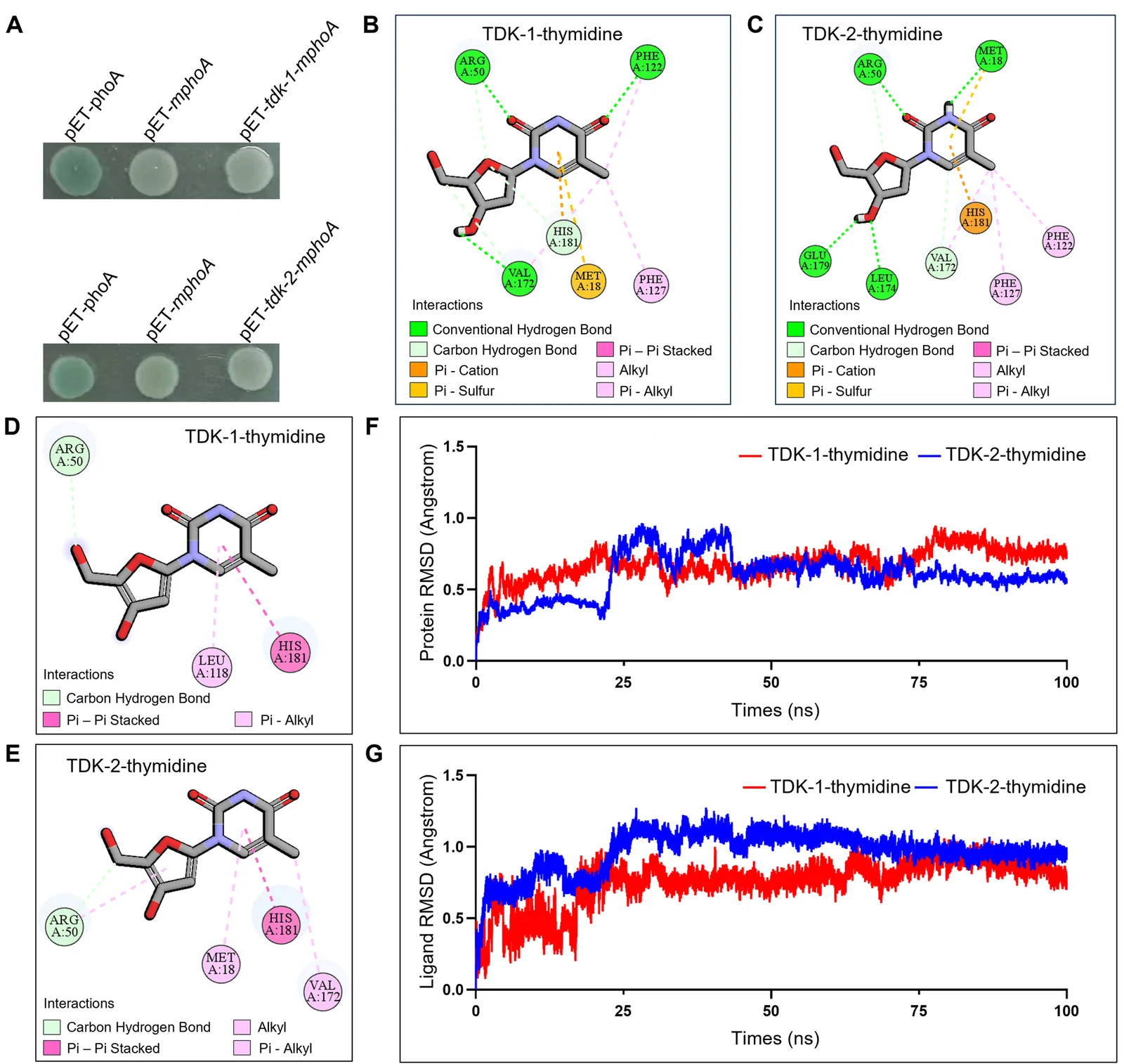
In silico analysis of the function of PaWB TDK. **A** PaWB TDK lacks extracellular transport activity. The positive control (*Escherichia coli* harboring pET-phoA plasmid) turned blue on the indicator medium. In contrast, the test strains (carrying pET-*tdk-1*-mphoA or pET-*tdk-2*-mphoA plasmids) were white, consistent with the negative control (*E. coli* with pET-mphoA plasmid). **B** and **C** Molecular docking models of TDK-1 and TDK-2 with thymidine predicted with AutoDock Vina (version 1.1.2). **D** and **E** Final-frame interaction modes of TDK-1/TDK-2 with thymidine from equilibrated molecular dynamics simulations predicted with GROMACS (version 2023.3). **F** Time evolution of the protein root mean square deviation (RMSD) for TDK-1-thymidine and TDK-2-thymidine complexes. **G** Time evolution of ligand RMSD for TDK-1-thymidine and TDK-2-thymidine complexes

PaWB TDK-1 and TDK-2 were both 209-amino acid proteins with a molecular weight of 23.34 kDa. Cell-PLoc prediction indicated that TDK localized to the cytoplasm (Fig. S2). SignalP 5.0 predicted no signal peptides in TDK-1 or TDK-2 (Fig. S3), which was demonstrated in the *E*. *coli* secretion assays (Fig. 3A). NCBI CD analysis showed that TDK-1 and TDK-2 belong to the thymidine kinase superfamily (Fig. S4). Furthermore, molecular docking with AutoDock Vina (v1.1.2) following AlphaFold3-based structural modeling revealed distinct thymidine-binding modes in PaWB TDK-1 and TDK-2 (Fig. 3B, C). Molecular dynamics simulation revealed that the interactions of TDK-1 and TDK-2 with thymidine in the final equilibrated frame showed different dynamic behaviors (Fig. 3D, E). The TDK-2-thymidine complex exhibited higher root mean square deviation fluctuations in both the protein and ligand during equilibration than TDK-1-thymidine (Fig. 3F, G), indicating its lower structural stability. These results suggest that TDK-1 and TDK-2 have different enzymatic functions.

### TDK is expressed in PaWB-infected tissues and co-localizes with PaWB in sieve tube elements

**Fig. 4:**
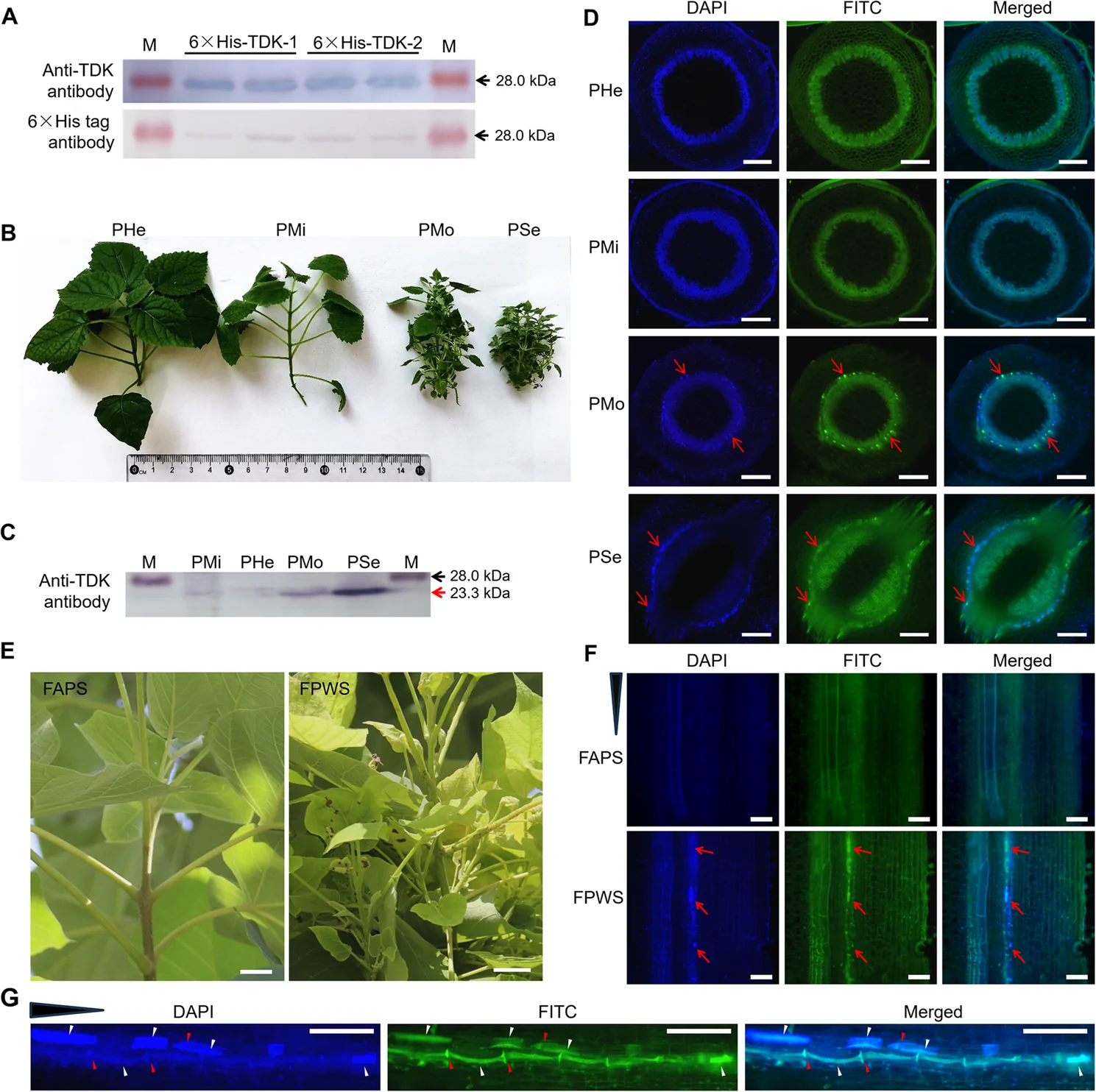
‌Protein expression and immunofluorescence microscopy (IFM) analysis of PaWB TDK in infected tissues. **A** Detection of the custom-generated anti-TDK polyclonal antibody. Western blot analysis using anti-His antibody confirms the presence and correct size (~ 27 kDa) of the recombinant 6×His-TDK-1 and 6×His-TDK-2 proteins. Full-length blots are presented in Supplementary Figure S6. **B** Disease symptoms in tissue-cultured paulownia plantlets. Phe, Healthy control; PMi, Mild symptoms; PMo, Moderate symptoms; PSe, Severe symptoms. **C** Western blot analysis of Phe, PMi, PMo, and PSe using anti-TDK polyclonal antibody. The black arrow indicates the 28 kDa protein marker band, and the red arrow indicates the size of the TDK protein. M, protein marker. Full-length blots are presented in Supplementary Figure S7. **D** IFM detection of PaWB TDK expression in paulownia plantlets. IFM images show the transverse sections of the stem segment. Red arrows indicate the phloem regions showing co-localization of DAPI (blue) and FITC (green) fluorescent signals. Scale bar = 300 μm. **E** Field paulownia samples. FAPS, field-grown asymptomatic paulownia sample. FPWS, field-grown PaWB sample. Scale bar = 1 cm. **F** IFM detection of PaWB TDK expression in field paulownia samples. IFM images show the longitudinal sections of the stem segment. Red arrows indicate the phloem regions showing co-localization of DAPI (blue) and FITC (green) fluorescent signals. Scale bar = 300 μm. **G** Expression of the TDK protein in sieve tube elements of infected samples. White triangles indicate sieve tube elements; red triangles indicate sieve plates; black triangles show the direction from morphological apex to the base of the stem. Scale bar = 300 μm

The anti-TDK polyclonal antibody was generated using purified recombinant TDK protein to analyze TDK protein expression (Figs. 4A, S5). Tissue-cultured plantlets with different severities of PaWB disease-mild (PMi), moderate (PMo), and severe (PSe) (Fig. 4B)-were used to detect TDK expression. Healthy plantlets (PHe) served as a negative control (Fig. 4B). TDK protein expression was detected in PMi, PMo, and PSe, but not in PHe (Fig. 4C). Immunofluorescence microscopy indicated co-localization of FITC (TDK) and DAPI (phytoplasma) signals in the phloem of PMo and PSe samples (Fig. 4D). No signal was detected in PMi, which may be due to sub-threshold pathogen levels (Fig. 4D). Similar co-localization of TDK and the phytoplasma was observed in field-infected samples (Fig. 4E, F). Furthermore, IFM images of infected phloem samples showed that FITC and DAPI fluorescence co-localized in the sieve tube elements (Fig. 4G). Overall, these results demonstrate that TDK is expressed in PaWB-infected tissues and co-localizes with PaWB in the sieve tube elements.

### TDK expression is positively associated with phytoplasma proliferation

**Fig. 5:**
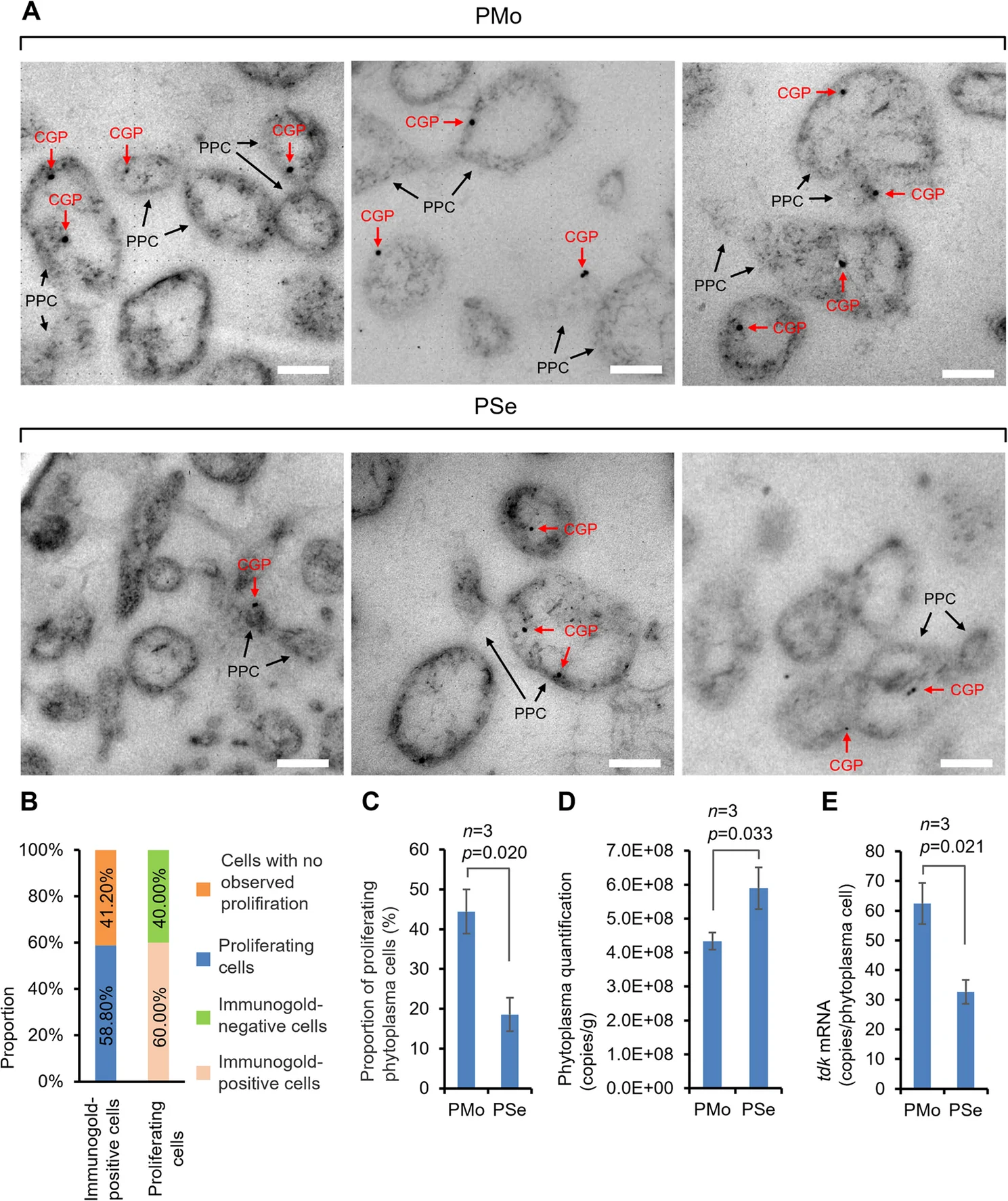
‌Subcellular localization of PaWB TDK by immunoelectron microscopy and *tdk* mRNA expression analysis. **A** Immunoelectron micrographs of PSe and PMi. CGP, colloidal gold particles; PPC, proliferating phytoplasma cells. Scale bar = 200 nm. **B** Morphometric proportion analysis of phytoplasma cells. **C** Proportion of proliferating phytoplasma cells. **D** Quantification of phytoplasmas by absolute qPCR. **E** Absolute quantification of PaWB *tdk* mRNA. In panels **C**-**E**, statistical analyses were performed with Student’s *t*-test. Bars indicate mean ± standard error (*n* = 3)

We used immunoelectron microscopy (IEM) to determine the localization of TDK in the phytoplasma cells of PMo and PSe samples. Colloidal gold particles showed that TDK expression localized in the cytosol of the phytoplasmas (Fig. 5A). Cell counting based on IEM images revealed that among the proliferating cells in PMo and PSe, 60% were immunogold positive, and 40% were immunogold negative (Fig. 5B). Of all immunogold-labeled cells, 58.8% were proliferating and 41.2% were non-proliferating (Fig. 5B). These results suggest that TDK expression is associated with proliferating phytoplasma cells.

Cell counting revealed that the proportion of proliferating cells was significantly higher in PMo than in PSe (Fig. 5C, *p* < 0.05), indicating a higher phytoplasma proliferation rate in PMo. Absolute quantitative PCR showed that the phytoplasma copy number per gram of sample was significantly lower in PMo than in PSe (Fig. 5D, *p* < 0.05). In contrast, the *tdk* mRNA copy number per phytoplasma was significantly higher in PMo than in PSe (Fig. 5E, *p* < 0.05). These results indicate that the expression of the *tdk* gene is positively associated with the phytoplasma proliferation rate.

### TDK promotes disease development by facilitating pathogen bacterial proliferation

**Fig. 6:**
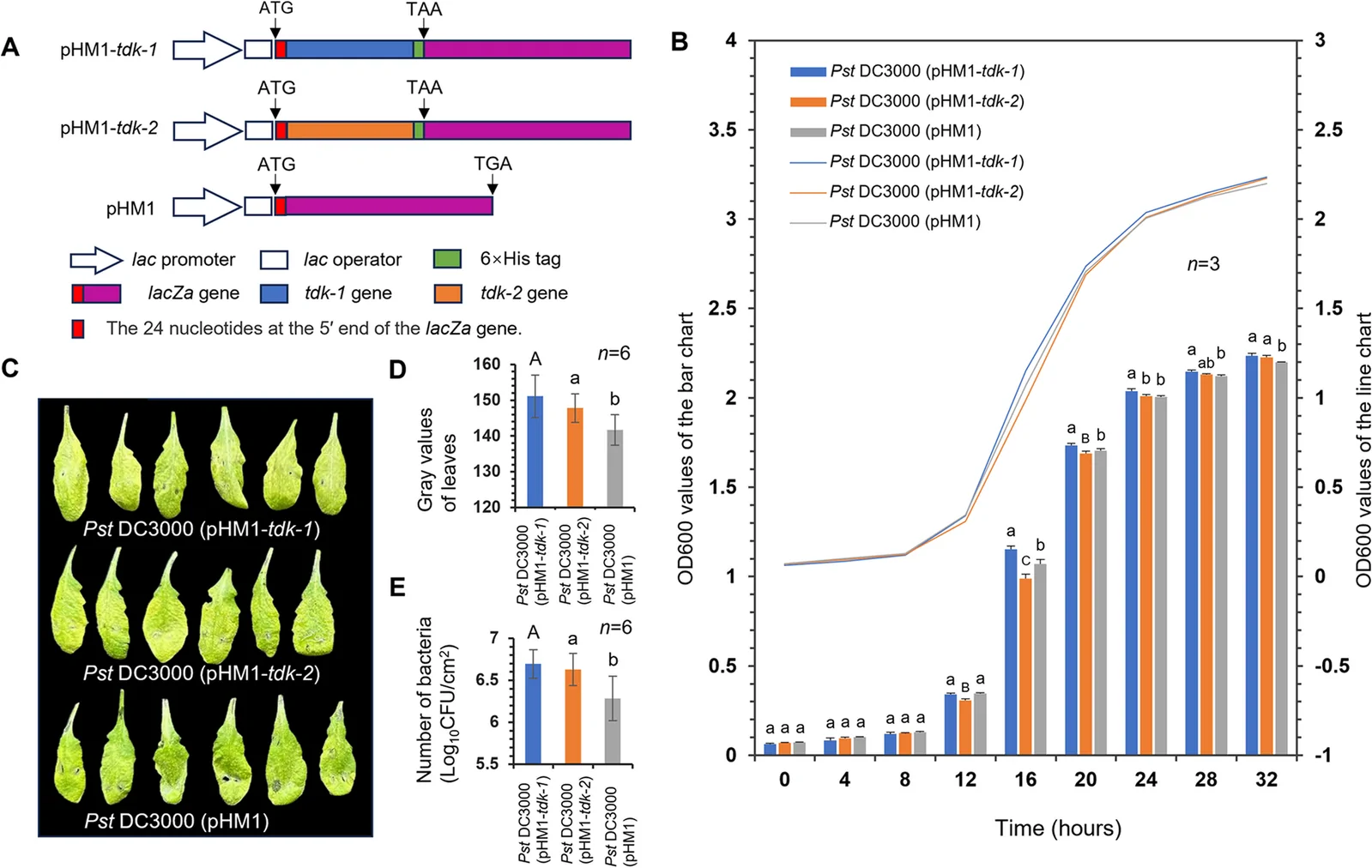
PaWB TDK promotes *Pseudomonas syringae* pv. *tomato* (*Pst*) DC3000 proliferation and disease severity. **A** Schematic diagram of plasmid constructs: pHM1-*tdk-1* and pHM1-*tdk-2*. **B** Growth curves of strains *Pst* DC3000 (pHM1-*tdk-1*), *Pst* DC3000 (pHM1-*tdk-2*), and *Pst* DC3000 (pHM1). Bacterial cultures were sampled every 4 h, and the OD_600_ was measured to generate growth curves. Statistical analyses were performed among the three experimental groups at each time point (one-way ANOVA with Tukey’s test). In the bar graphs, data are presented as the mean ± standard error from three biological replicates. Groups labeled with different lowercase letters differ significantly (*p* < 0.05), and those with different lowercase and uppercase letters differ highly significantly *(p* < 0.01). Groups sharing identical letters show no statistically significant difference. The experiments were repeated three times with similar results. **C** Phenotypes of *Arabidopsis thaliana* infected with *Pst* DC3000 strains at 3 days post-infiltration (dpi). **D** PaWB TDK promotes disease symptom development. The severity of *Pst* DC3000-induced chlorotic symptoms in *A. thaliana* was measured by 8-bit grayscale image analysis, with the grayscale intensity serving as a quantitative disease index. **E** PaWB TDK promotes *Pst* DC3000 colonization. Bacterial colonization was determined as colony-forming units (CFU/cm^2^) at 3 dpi. Bars represent the mean *±* standard error of three biological replicates (six leaves per sample). The experiments were repeated three times with similar results

Given the uncultivability of phytoplasmas, we employed *Pst* DC3000 and *Arabidopsis thaliana* to determine the ability of TDK to promote disease. Recombinant plasmids (pHM1-*tdk-1* and pHM1-*tdk-2*) and an empty vector control were introduced into *Pst* DC3000 (Fig. 6A). We analyzed the bacterial growth curves of both recombinant strains and found that the growth curves all started with the same density at inoculation (0 h). However, *Pst* strains containing pHM1-*tdk-1* significantly outperformed the control by 16 h (*p* < 0.01), and *Pst* strains containing pHM1-*tdk-2* exceeded the control until 32 h (*p* < 0.05) (Fig. 6B). A similar growth-promoting effect was observed in *E. coli* BL21(DE3) (Fig. S8), indicating the conserved function of both TDK homologs, with TDK-1 acting more rapidly and strongly.

To determine the ability of *tdk* to promote disease, *Arabidopsis* leaves were inoculated with *Pst* DC3000 carrying pHM1-*tdk-1*, pHM1-*tdk-2*, or the empty vector pHM1. Leaves inoculated with *Pst* strains containing either pHM1-*tdk-1* or pHM1-*tdk-2* exhibited more severe chlorosis compared to the control at 3 days post-infiltration (dpi) (Fig. 6C, D). Moreover, bacterial colonization of *Pst* strains carrying pHM1-*tdk-1* or pHM1-*tdk-2* were significantly higher than those of the control at 3 dpi (Fig. 6E). Overall, these results indicate that TDK-1 and TDK-2 enhance bacterial proliferation and disease progression.

## Discussion

Phytoplasmas, which are obligate parasites that colonize phloem sieve tubes in host plants and insect vectors [1], have highly reduced genomes with minimal biosynthetic capabilities [35], driving their continuous adaptive genetic variation [36]. Kong et al. identified two major phylogenetic lineages of PaWB phytoplasma based on housekeeping gene (*rp*, *fusA*, *secY*, *tuf*, *secA*, *dnaK*, *rpoB*, *pyrG*, *gyrB*, *ipt*) analysis of 142 strains across 18 provinces and municipalities in China: lineage-1, a widespread and dominant population, and lineage-2, restricted to Jiangxi, Fujian, and Shaanxi provinces [6]. In our nationwide survey of 87 strains from 15 provinces or municipalities, two *tdk* genotypes were detected—*tdk-1*, broadly distributed, and *tdk-2*, exclusively found in Jiangxi, Fujian, and Shaanxi (Fig. 1)—showing near-perfect concordance with the two lineages. Notably, lineage-2 and *tdk-2* were predominantly localized in Fujian and Jiangxi, with only a single strain detected in Shaanxi, mirroring the spatial pattern reported by Kong et al. [6]. This genetic-geographic correlation supports the Kong’s hypothesis that lineage-2 divergence may have arisen through long-term geographic isolation, host-specific adaptation, or clonal resistance [6].

The intracellular dNTP pool governs cell cycle progression and proliferation [37]. A previous study in *E. coli* showed that precise modulation of ribonucleotide reductase (RNR) expression, the key enzyme that reduces ribonucleotides to deoxyribonucleotides, lowered the dNTP concentration, delaying the cell cycle and slowing proliferation [38]. We observed a comparable phenomenon in which PaWB *tdk* overexpression in *Pst* DC3000 and *E. coli* BL21 (DE3) significantly enhanced bacterial proliferation (Figs. 6B, S8). Stable enzyme-substrate binding is essential for catalytic activity, as reflected by low RMSD fluctuations in molecular dynamics simulations [39]. Molecular dynamics simulations of PaWB TDK–substrate interactions indicated that the TDK-1-thymidine complex had greater structural stability than the TDK-2-thymidine complex (Fig. 3F, G), suggesting that TDK-1 has a higher catalytic efficiency. Thus, the sequence variations between TDK-1 and TDK-2, particularly the amino acid substitutions at positions 2 (Ser/Thr) and 199 (Ile/Val), could represent adaptive mutations that optimize the enzymatic function under different ecological contexts. Future studies will further analyze the enzymatic activities of TDK-1 and TDK-2 to better explain the biological importance of the sequence differences and their growth-promoting efficiency.

To thrive in nature, bacteria must rapidly proliferate under favorable conditions but persist under stress [40]. The enrichment of TDK expression in proliferating cells, as demonstrated by IEM (Fig. 5A), underscores its stage-specific role in the life history of phytoplasmas. Besides, functional validation in *Pseudomonas syringae* suggests the adaptive value of TDK-mediated proliferation (Fig. 6). Previous studies reported that aster yellows phytoplasma adapt to the diverse environments of their plant and insect hosts by altering gene expression [41]. These findings align with evolutionary trends in reduced-genome pathogens, in which efficient nutrient salvage is essential for survival [5, 42]. However, unlike phytoplasmas that rely exclusively on the salvage pathway for dTTP production, *Pst* DC3000 possesses a complete *de novo* synthesis pathway. Thus, the heterologous system tests whether TDK confers an additional growth advantage even when the *de novo* pathway is operational. Future measurements of dTTP levels are needed to directly link TDK expression to dTTP pool expansion. Although heterologous overexpression in *P. syringae* provides functional evidence for a growth-promoting role of TDK, the precise contribution of TDK to phytoplasma proliferation in its native context remains to be directly validated once phytoplasma genetic manipulation systems become available.

Nucleotide biosynthesis is the biochemical foundation of bacterial pathogenicity [43]. Pathogenic bacteria with impaired nucleotide synthesis have attenuated virulence [44, 45], whereas the overexpression of nucleotide biosynthesis-related genes enhances bacterial pathogenicity [21, 22]. Premature mutations in pyrR, a key regulator of pyrimidine synthesis, in methicillin-resistant *S. aureus* cause the overexpression of pyrimidine pathway genes, conferring advantages in gastrointestinal colonization and accelerating the progression of infection [22]. Our study revealed an analogous mechanism in PaWB phytoplasmas, in which the overexpression of PaWB *tdk* enhanced the pathogenicity of *Pst* DC3000 (Fig. 6). Unlike canonical secreted phytoplasma effectors such as SAP11 or TENGU [46, 47], TDK does not directly manipulate phytoplasma pathogenicity but instead promotes phytoplasma proliferation, thereby increasing bacterial titer. Since witches’ broom symptoms only manifest when phytoplasma load exceeds a critical threshold [10], TDK-mediated multiplication may serve as a prerequisite for effectors to accumulate to effective concentrations. Thus, we infer that TDK acts indirectly and synergistically: it elevates pathogen burden, enabling effectors to reach the threshold required for symptom induction.

Quorum sensing (QS) is a bacterial intercellular communication system involving the synthesis and secretion of signaling molecules [48–51]. These molecules accumulate with cell density and, upon reaching a threshold, bind intracellular receptors to regulate gene expression [52]. QS inhibits nucleic acid synthesis in pathogenic bacteria by downregulating purine and pyrimidine biosynthesis genes [53–57]. In our study, *tdk* transcript levels were negatively associated with phytoplasma proliferation rate (Fig. 5D, E), raising the possibility that a density-dependent regulatory mechanism analogous to QS may control *tdk* expression. Whether this involves QS regulation remains to be investigated, a question that may open new avenues in phytoplasma research.

## Conclusion

**Fig. 7:**
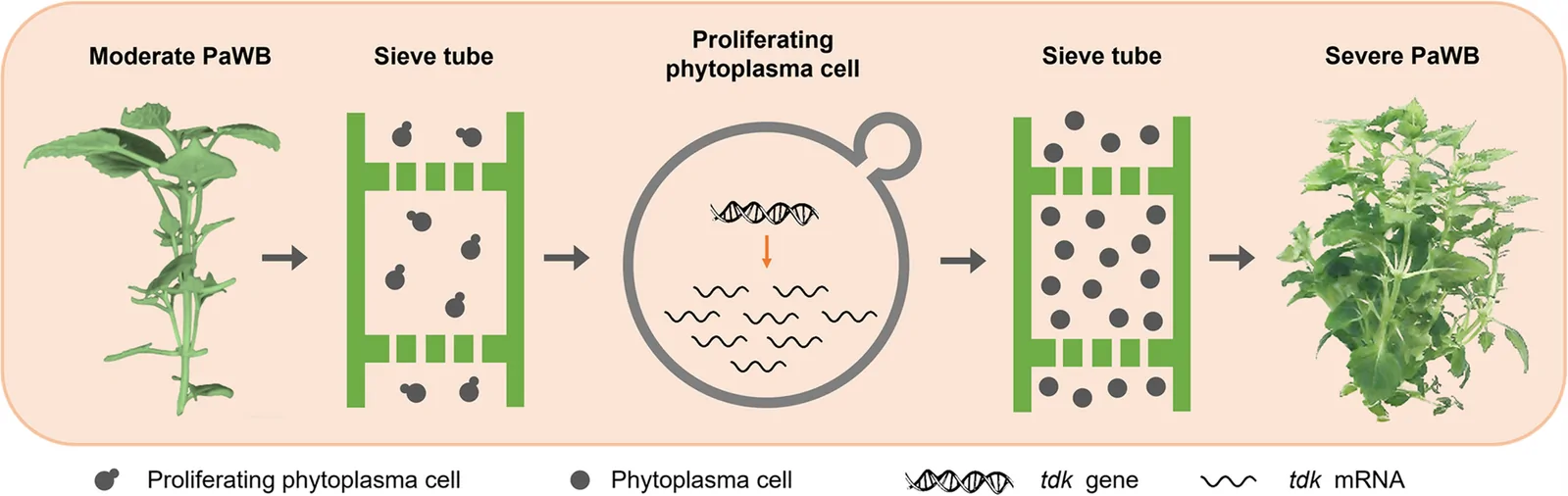
Proposed working model showing that the *tdk* gene promotes symptom aggravation by enhancing phytoplasma proliferation. During the early stage of pathogen infection, when phytoplasma titers are low in the host, the expression of phytoplasma *tdk* gene mRNA is upregulated. This increased mRNA expression promotes phytoplasma proliferation, resulting in a higher pathogen load and the exacerbation of disease symptoms

The study revealed that the *tdk-1* genotype of PaWB phytoplasmas was more widely distributed than *tdk-2*, and its protein exhibited superior substrate-binding stability—suggesting functional optimization through genetic variation. We demonstrated that phytoplasma TDK promoted the proliferation and virulence of *Pst* DC3000, supporting our model in which phytoplasmas regulate their pathogenicity and established TDK as a key proliferation-associated pathogenicity factor. Although direct functional validation in phytoplasmas remains technically unfeasible, heterologous expression provides robust functional evidence. Furthermore, we observed a negative relationship between the phytoplasma load and *tdk* mRNA levels per cell during symptom progression, indicating density-dependent transcriptional control. This opens a new avenue for investigating population-level signaling mechanisms in phytoplasmas. In summary, our findings show that TDK-mediated nucleotide salvage enhances phytoplasma proliferation and symptom severity, identifying it as a key adaptive mechanism for pathogenicity (Fig. 7). The present study establishes a rationale for controlling phytoplasma diseases through targeting TDK.

## Supplementary Information

Supplementary Material 1.

## Data Availability

The nucleotide sequences of the *tdk-1* and *tdk-2* genes from the PaWB phytoplasmas have been deposited in GenBank under accession numbers OP913234 and OP913235, respectively. All data generated or analyzed during this study are included in this published article and its supplementary information files.

## References

[1] Wang R, Bai B, Li D, Wang J, Huang W, Wu Y, Zhao L. Phytoplasma: a plant pathogen that cannot be ignored in agricultural production-research progress and outlook. Mol Plant Pathol. 2024;25(2):e13437. 10.1111/mpp.13437. PMC10887288 38393681

[2] Fan GQ. Epigenetics of paulownia witches’ broom. Beijing: Science; 2022.

[3] G.Q. Fan. Modern genetics and breeding of paulownia plants, Science. Beijing. 2024.

[4] Guo N, Zhai XQ, Fan GQ. Chemical composition, health benefits and future prospects of paulownia flowers: a review, Food Chem. 412 (2023) 135496. 10.1016/j.foodchem.2023.135496. 36720182

[5] Zhang Y, Qiao Z, Li J, Bertaccini A. Paulownia witches’ broom disease: a comprehensive review. Microorganisms. 2024;12:885. 10.3390/microorganisms12050885. PMC11123829 38792713

[6] Kong DZ, Lin CL, Yu SS, Tian GZ, Ma HB, Wang SJ. Molecular diversity and evolutionary relatedness of paulownia witches’-broom phytoplasma in different geographical distributions in China, Biology (Basel). 2022;11(11):1611. 10.3390/biology11111611. PMC9687790 36358312

[7] J.P. Jiang. Paulownia cultivation, China Forestry Publishing House. Beijing. 1990.

[8] Ren GL. Present research state and development of paulownia witches’ broom. J Henan Agric Univ. 1996;30(4):358–64. 10.16445/j.cnki.1000-2340.1996.04.012.

[9] Carreón-Anguiano KG, Vila-Luna SE, Sáenz-Carbonell L. Canto-Canché. Novel Insights into Phytoplasma Effectors. Horticulturae. 2023;9(11):1228. 10.3390/horticulturae9111228.

[10] Ren ZG, Wang H, Lin CL, Liu X, Song CS, Feng SK, Yu SS, Lu XL, Tian GZ. A real-time (SYBR Green I) PCR assay for detection and quantification of jujube witches’-broom phytoplasma in the grafted jujube cultivar scions with different resistance. Acta Phytopathol Sin. 2015;45(5):520–9. 10.13926/j.cnki.apps.2015.05.010.

[11] Oshima K, Maejima K, Namba S. Genomic and evolutionary aspects of phytoplasmas. Front Microbiol. 2013;4:230. 10.3389/fmicb.2013.00230. PMC3743221 23966988

[12] Tao J, Wang Z, Shi R, Lin L, Li M, Meng Y, Luo S, Jiang X, Guo Z, Shang Y, Lu Z. ERK-USP9X-coupled regulation of thymidine kinase 1 promotes both its enzyme activity-dependent and its enzyme activity-independent functions for tumor growth. Nat Struct Mol Biol. 2025;32(5):853–63. 10.1038/s41594-024-01473-6. 39824978

[13] Chen YL, Eriksson S, Chang ZF. Regulation and functional contribution of thymidine kinase 1 in repair of DNA damage. J Biol Chem. 2010;285(35):27327–35. 10.1074/jbc.M110.137042. PMC2930731 20554529

[14] Bitter EE, Townsend MH, Erickson R, Allen C, O’Neil KL. Thymidine kinase 1 through the ages: a comprehensive review. Cell Biosci. 2020;10(1):138. 10.1186/s13578-020-00493-1. PMC7694900 33292474

[15] Li Z, Wu Z, You X, Tang N. Pan-cancer analysis reveals that TK1 promotes tumor progression by mediating cell proliferation and Th2 cell polarization, Cancer Cell Int. 2024;24(1):329. 10.1186/s12935-024-03515-x. PMC11440694 39343871

[16] Bello LJ. Regulation of thymidine kinase synthesis in human cells. Exp Cell Res. 1974;89(2):263–74. 10.1016/0014-4827(74)90790-3. 4457349

[17] Jagarlamudi KK, Shaw M. Thymidine kinase 1 as a tumor biomarker: technical advances offer new potential to an old biomarker. Biomark Med. 2018;12(9):1035–48. 10.2217/bmm-2018-0157. 30039979

[18] Goel S, Bergholz JS, Zhao JJ. Targeting CDK4 and CDK6 in cancer. Nat Rev Cancer. 2022;22:356–72. 10.1038/s41568-022-00456-3. PMC9149100 35304604

[19] Clausen AR, Girandon L, Ali A, Knecht W, Rozpedowska E, Sandrini MPB, Andreasson E, Munch-Petersen B, Piškur J. Two thymidine kinases and one multisubstrate deoxyribonucleoside kinase salvage DNA precursors in A*rabidopsis thaliana*, FEBS J. 2012;279(20):3889–3897. 10.1111/j.1742-4658.2012.08747.x. 22897443

[20] Xie Y, Wu L, Wang M, Cheng A, Yang Q, Wu Y, Jia R, Zhu D, Zhao X, Chen S, Liu M, Zhang S, Wang Y, Xu Z, Chen Z, Zhu L, Luo Q, Liu Y, Yu Y, Zhang L, Chen X. Alpha-herpesvirus thymidine kinase genes mediate viral virulence and are potential therapeutic targets. Front Microbiol. 2019;10:941. 10.3389/fmicb.2019.00941. PMC6517553 31134006

[21] Goncheva MI, Flannagan RS, Sterling BE, Laakso HA, Friedrich NC, Kaiser JC, Watson DW, Wilson CH, Sheldon JR, McGavin MJ, Kiser PK, Heinrichs DE. Stress-induced inactivation of the *Staphylococcus aureus* purine biosynthesis repressor leads to hypervirulence. Nat Commun. 2019;10(1):775. 10.1038/s41467-019-08724-x. PMC6377658 30770821

[22] Copin, Sause WE, Fulmer Y, Balasubramanian D, Dyzenhaus S, Ahmed JM, Kumar K, Lees J, Stachel A, Fisher JC, Drlica K, Phillips M, Weiser JN, Planet PJ, Uhlemann AC, Altman DR, Sebra R, van Bakel H, Lighter J, Torres VJ, Shopsin B. Sequential evolution of virulence and resistance during clonal spread of community-acquired methicillin-resistant *Staphylococcus aureus*, Proc. Natl. Acad. Sci. USA. 2019;116(5):1745–54. 10.1073/pnas.1814265116. PMC6358666 30635416

[23] Kumar S, Stecher G, Tamura K. Mega7: molecular evolutionary genetics analysis version 7.0 for bigger datasets. Mol Biol Evol. 2016;33(7):1870–4. 10.1093/molbev/msw054. PMC8210823 27004904

[24] Saitou N, Nei M. The neighbor-joining method: a new method for reconstructing phylogenetic tree, Mol. Biol. Evol. 1987;4(4):406–425. 10.1093/oxfordjournals.molbev.a040454. 3447015

[25] Felsenstein J. Confidence limits on phylogenies: an approach using the bootstrap. Evolution. 1985;39(4):783–91. 10.1111/j.1558-5646.1985.tb00420.x. 28561359

[26] Xie J, Chen Y, Cai G, Cai R, Hu Z, Wang H. Tree visualization by one table (tvBOT): a web application for visualizing, modifying and annotating phylogenetic trees. Nucleic Acids Res. 2023;55(W1):W587–92. 10.1093/nar/gkad359. PMC10320113 37144476

[27] Wilkins MR, Gasteiger E, Bairoch A, Sanchez JC, Williams KL, Appel RD, Hochstrasser DF. Protein identification and analysis tools in the ExPASy server. Methods Mol Biol. 1999;112:531–52. 10.1385/1-59259-584-7:531. 10027275

[28] Almagro Armenteros JJ, Tsirigos KD, Sønderby CK, Petersen TN, Winther O, Brunak S, von Heijne G, Nielsen H. SignalP 5.0 improves signal peptide predictions using deep neural networks. Nat Biotechnol. 2019;37(4):420–3. 10.1038/s41587-019-0036-z. 30778233

[29] Chou KC, Shen HB. Cell-PLoc: a package of Web servers for predicting subcellular localization of proteins in various organisms. Nat Protoc. 2008;3(2):153–62. 10.1038/nprot.2007.494. 18274516

[30] Wan CP, He FX, Zhang W, Xu Q, Zhu QL, Song CS. A sec-dependent effector from Candidatus phytoplasma ziziphi suppresses plant immunity and contributes to pathogenicity. Biology (Basel). 2025;14(5):528. 10.3390/biology14050528. PMC12108763 40427717

[31] Trott O, Olson AJ. AutoDock Vina: improving the speed and accuracy of docking with a new scoring function, efficient optimization, and multithreading. J Comput Chem. 2010;31(2):455–61. 10.1002/jcc.21334. PMC3041641 19499576

[32] Ji X, Gai Y, Lu B, Zheng C, Mu Z. Shotgun proteomic analysis of mulberry dwarf phytoplasma. Proteome Sci. 2010;8:20. 10.1186/1477-5956-8-20. PMC2873370 20377883

[33] Shi R, Liu F, Huo G, Yang L. Real-time RT-PCR based on DNA subtraction for absolute quantification of gene expression in engineered lactic acid bacteria. Chin J Biotechnol. 2009;25(8):1240–6. 10.3321/j.issn:0001-6209.2008.04.019. 19938463

[34] An Y, Chen J, Xu Z, Ouyang X, Cao P, Wang R, Liu P, Zhang M. Three amino acid residues are required for the recognition of *Ralstonia solanacearum* RipTPS in *Nicotiana tabacum*, Front. Plant Sci. 2022;13:1040826. 10.3389/fpls.2022.1040826. PMC9606615 36311066

[35] Oshima K, Kakizawa S, Nishigawa H, Jung HY, Wei W, Suzuki S, Arashida R, Nakata D, Miyata S, Ugaki M, Namba S. Reductive evolution suggested from the complete genome sequence of a plant-pathogenic phytoplasma. Nat Genet. 2004;36(1):27–9. 10.1038/ng1277. 14661021

[36] Bleuven C, Landry CR. Molecular and cellular bases of adaptation to a changing environment in microorganisms. Proc Biol Sci. 2016;283(1841):20161458. 10.1098/rspb.2016.1458. PMC5095378 27798299

[37] Mathews CK. Deoxyribonucleotides as genetic and metabolic regulators. FASEB J. 2014;28(9):3832–40. 10.1096/fj.14-251249. PMC5395721 24928192

[38] Zhu M, Dai X, Guo W, Ge Z, Yang M, Wang H, Wang YP. Manipulating the bacterial cell cycle and cell size by titrating the expression of ribonucleotide reductase, mBio. 2017;8(6):e01741–17. 10.1128/mBio.01741-17. PMC5686538 29138305

[39] Pavan M, Menin S, Bassani D, Sturlese M, Moro S. Qualitative estimation of protein-ligand complex stability through thermal titration molecular dynamics simulations. J Chem Inf Model. 2022;62(22):5715–28. 10.1021/acs.jcim.2c00995. PMC9709921 36315402

[40] Zhu M, Dai X. Shaping of microbial phenotypes by trade-offs. Nat Commun. 2024;15:4238. 10.1038/s41467-024-48591-9. PMC11102524 38762599

[41] Makarova O, MacLean AM. Nicolaisen. Phytoplasma adapt to the diverse environments of their plant and insect hosts by altering gene expression. Physiol Mol Plant Pathol. 2015;91:81–7. 10.1016/j.pmpp.2015.06.003.

[42] Miyata SI, Oshima K, Kakizawa S, Nishigawa H, Jung HY, Kuboyama T, Ugaki M. S. Namba. Two different thymidylate kinase gene homologues, including one that has catalytic activity, are encoded in the onion yellows phytoplasma genome, Microbiology. 2003;149(Pt 8):2243–2250. 10.1099/mic.0.25834-0. 12904564

[43] Goncheva MI, Chin D, Heinrichs DE. Nucleotide biosynthesis: the base of bacterial pathogenesis, Trends Microbiol. 2022;30(8):793–804. 10.1016/j.tim.2021.12.007. 35074276

[44] Goncheva MI, Flannagan RS, Heinrichs DE. *De novo* purine biosynthesis is required for intracellular growth of *staphylococcus aureus* and for the hypervirulence phenotype of a *purR* mutant, Infect Immun. 2020;88(5)e00104–20. 10.1128/IAI.00104-20. PMC7171247 32094249

[45] Lan L, Cheng A, Dunman PM, Missiakas D, He C. Golden pigment production and virulence gene expression are affected by metabolisms in *Staphylococcus aureus*, J. Bacteriol. 2010;192(12):3068–3077. 10.1128/JB.00928-09. PMC2901709 20400547

[46] Sugio A, Kingdom HN, MacLean AM, Hogenhout SA. Phytoplasma protein effector SAP11 enhances insect vector reproduction by manipulating plant development and defense hormone biosynthesis, Proc. Natl. Acad. Sci. USA. 2011;108(48):E1254–E1263. 10.1073/pnas.1105664108. PMC3228479 22065743

[47] Hoshi A, Oshima K, Kakizawa S, Ishii Y, Ozeki J, Hashimoto M, Komatsu K, Kagiwada S, Yamaji Y, Namba S. A unique virulence factor for proliferation and dwarfism in plants identified from a phytopathogenic bacterium, Proc. Natl. Acad. Sci. USA. 2009;106(15):6416–6421. 10.1073/pnas.0813038106. PMC2669400 19329488

[48] Nealson KH, Platt T, Hastings JW. Cellular control of the synthesis and activity of the bacterial luminescent system, J. Bacteriol. 1970;104(1):313–322. 10.1128/jb.104.1.313-322.1970. PMC248216 5473898

[49] Fuqua WC, Winans SC, Greenberg EP Quorum sensing in bacteria: the LuxR-LuxI family of cell density-responsive transcriptional regulators, J. Bacteriol. 1994;176(2):269–275. 10.1146/annurev.genet.35.102401.090913. PMC205046 8288518

[50] Fuqua C, Parsek MR, Greenberg EP. Regulation of gene expression by cell-to-cell communication: acyl-homoserine lactone quorum sensing, Annu. Rev. Genet. 2001;35:439–468. 10.1146/annurev.genet.35.102401.090913. 11700290

[51] Miller MB, Bassler BL. Quorum sensing in bacteria, Annu. Rev. Microbiol. 2001;55:165–199. 10.1146/annurev.micro.55.1.165. 11544353

[52] Jenul C, Sieber S, Daeppen C, Mathew A, Lardi M, Pessi G, Hoepfner D, Neuburger M, Linden A, Gademann K. L. Eberl. Biosynthesis of fragin is controlled by a novel quorum sensing signal, Nat. Commun. 2018;9(1):1297. 10.1038/s41467-018-03690-2. PMC5878181 29602945

[53] An JH, Goo E, Kim H, Seo YS, Hwang I. Bacterial quorum sensing and metabolic slowing in a cooperative population, Proc. Natl. Acad. Sci. USA. 2014;111(41):14912–14917. 10.1073/pnas.1412431111. PMC4205598 25267613

[54] Majerczyk C, Brittnacher M, Jacobs M, Armour CD, Radey M, Schneider E, Phattarasokul S, Bunt R, Greenberg EP. Global analysis of the *Burkholderia thailandensis* quorum sensing-controlled regulon, J. Bacteriol. 2014;196(7):1412–1424. 10.1128/JB.01405-13. PMC3993349 24464461

[55] Goo E, An JH, Kang Y, Hwang I. Control of bacterial metabolism by quorum sensing, Trends Microbiol. 2015;23(9):567–576. 10.1016/j.tim.2015.05.007. 26072043

[56] Yan C, Li X, Zhang G, Zhu Y, Bi J, Hao H, Hou H. Quorum sensing-mediated and growth phase-dependent regulation of metabolic pathways in *Hafnia alvei* H4, Front. Microbiol. 2021;12:567942. 10.3389/fmicb.2021.567942. PMC7960787 33737914

[57] Zhang TZ, Liu LP, Ye L, Li WC, Xin B, Xie YY, Jia SR, Wang TF, Zhong C. The production of bacterial cellulose in *Gluconacetobacter xylinus* regulated by luxR overexpression of quorum sensing system, Appl. Microbiol. Biotechnol. 2021;105(20):7801–7811. 10.1007/s00253-021-11603-5. 34581846

